# Supplementary material for: Mouth breathing, dry air, and low water permeation promote inflammation, and activate neural pathways, by osmotic stresses acting on airway lining mucus
Source: QRB Discov. 2023 Feb 14;4:e3. doi: 10.1017/qrd.2023.1 (PMC10392678; doi:10.1017/qrd.2023.1)
Supplement: Supplementary file 1 [file S2633289223000017sup001.pdf]

Supplemental Material

Mouth breathing, dry air, and low water permeation promote inflammation, and activate neural pathways, by osmotic stresses acting on airway lining mucus

David A. Edwards[1]* and Kian Fan Chung[2]

[1]John Paulson School of Engineering & Applied Sciences, Harvard University, Cambridge, US
[2]Experimental Studies Unit, National Heart & Lung Institute, Imperial College London, UK

*Corresponding author: dedwards@seas.harvard.edu

We describe osmotic water flow within airway mucus over which water evaporates into warm, dry air laden with fine and ultra-fine particles. We determine leading-order analytical expressions for water flow and mucus/cilia compressive stress in the limit of warm air, and well hydrated airways, where the impact of heat and momentum transfer in the process of breath-activated water evaporation is dominated by osmotically-driven mass transfer. The model, and values determined for mucus water permeabilities and osmotic pressures, are based on an assumption of the airway mucus as a non-dehydrating (i.e. approximately perfectly hydrated) selective transport barrier, where extremely minute resistance of ion transport combined with the relatively thick transport barrier of the mucus, combine to produce a sensible osmotic water permeation across mucus to meet evaporative water flux needs. The roles in mucus transport of electrical current, mucus density evolution with drying or electric fields, or hindered diffusion of globular proteins and mucins, are not considered here. Particularly the scenario where water evaporation from above the mucus leads to water volume loss above the mucus, partial drying of the air-exposed mucus, and increase in mucus solids content, thereby generating osmolyte gradients across the mucus and resulting in a net force on the mucus pushing it toward cilia — is an alternative scenario to the following. The analysis assumes the perspective of a Lagrangian mucus observer, with an evaporative force pushing mucus against cilia — in contrast (while complementary) to the perspective of water being "pulled" into mucus pores consequent to drying and increase in solids content of the mucus, i.e. an "osmotic modulus" (Hill et al 2022).

Upper Airway Water Evaporation

Many previous efforts have clarified the processes of water evaporation that occur in human airways during the inhalation of environmental air (Haut et al 2021, Wu et al 2015, Ferron et al

1995). Our contribution is to note the impact of such evaporation on cilia stresses delivered to the airway epithelium notably in the upper airways by way of the transmission of osmotic pressure differential arising from water flow through airway mucus. We note in the following that the magnitude of these mass-transfer-delivered stresses relative to heat and momentum stresses is such that, at least in the context of the breathing of warm (30 C) air in well-hydrated airways, airway water evaporation can be understood to leading order purely in mass transfer terms.

The mass evaporative flux of water (Q_e) (kg/s) from the air/water interface over airway lining fluid and into airway lumen can be approximated per airway compartment (nose and trachea) of area A by the Penman Equation (MacArthur 1990)

$$Q_e \approx K_e A(x_s - x) \quad (1)$$

where x_s is the mass water per mass dry air at saturated conditions, x the value at actual air conditions, and the evaporation rate constant K_e (kg/m^2/h) per compartment (MacArthur 1990)

$$K_e \approx \left(\frac{1}{3600}\right)(25 + 19u_a) \quad (2)$$

including constant quiescent and convective evaporation contributions, the latter growing linearly with average compartmental air velocity u_a (m/s). We assume relatively quiescent conditions over the majority of surface area within the nose in that principal air flow occurs in the narrow air passage of the middle or inferior meatus, and an average velocity on inhalation in the trachea of around 1 m/s, dictated by the jet of air that forms on inhalation within the larynx (characteristic peak air velocity ~ 3 m/s), and that differentiates the tracheal evaporative mass transport conditions from the nose. The evaporation rate constant for the nose compartment follows as K_E ~ 0.007 kg/m^2/s, while for the tracheal compartment K_E ~ 0.01 kg/m^2/s. Standard humidity tables give x_s = 0.02 in the nose and tracheal compartments (ρ=1.225 kg/m^3), while x=0.002 at 10% RH and x=0.01 at 60% RH. This yields a total predicted average mass evaporative loss of water from the nose ranging from Q_e ~ 2 mg/s (10%RH) to Q_e ~ 1mg/s (60% RH), and in the trachea from Q_e ~ 1.2 mg/s (10%RH) to Q_e ~ 0.6 mg/s (60% RH). Assuming full condensation of super saturated water on exhalation at external air RH of 10% we estimate (see Tables 1 and 2) ~ 16.7 mg water condenses over the 220 cm^2 ALF surface in the upper airways,

leading to a condensation layer thickness of ~ 1 μm.   Over many inhalations and exhalations, a mean time-averaged osmotic flow rate of water through the mucus and the underlying epithelium can be determined by balancing this osmotic flux with time-averaged rate of evaporative water loss to the inhaled air.

Osmotic Pressure Acting on Airway Mucus

In its fully hydrated state, human airway mucus is a relatively permeable hydrogel with solids content of around 5% by weight, able to slow the movement of viruses, pathogens, and airborne particles of any kind, and immobilize particles of around 500 nm and larger (Walji 2010, Shuster et al 2013). Smaller size particles are also hindered in their movements, while in ways influenced by surface interactions with mucins, such that particles as small as 20 nm in diameter have been observed to be hindered in their diffusion relative to pure water (Walji 2010). Similar surface interactions also retard the movement of ions within mucus, albeit to weaker degree.

On the breathing of typical dirty air (PM 2.5 = 20 μg/m^3 and PM 0.1 = 20 μg/m^3) mucus in the upper airways becomes populated with deposited inhaled airborne particles. In dehydrating circumstances, this coverage naturally increases. Ultra-fine particles are known to predominately deposit in the upper airways on inhalation owing to Brownian motion (Cohen et al 1990). Assuming 50% of ultra-fine particle deposition in the trachea with an average diameter of ~ 20 nm, tidal breathing of air at tidal volume of 500 cm^3, 15 breaths per minute, upper airway mean mucus residence (clearance) times of either 2 hours (fully hydrated, cilia moving in the trachea at a rate of around 1 mm/min) or 24 hours (relatively dehydrated), it follows that between 10 μg to ~ 100 μg of ultra-fine particle mass exists over the surface of the tracheal airway lining fluid after an hour or so of breathing air with PM 0.1 equal to 20 μg/m^3. Similar and even greater masses of the larger particles (PM 2.5 and PM 10.0) will deposit in the trachea as well, recognizing that tracheal deposition of airborne particles is maximal for mass median particle diameters of ~ 10 μm owing to inertial impaction (Darquenne 2020). All of these particles will initially land on the surface of the mucus, and diffuse into the hydrogel with restricted movement conditioned by particle nature. Some of the larger particles will become trapped in the hydrogel, and this entrapment will reorient the smaller particles on their random walk through the hydrogel. Assuming a similar order of magnitude mass of PM 2.5 particles in the tracheal airway lining fluid, and given these particles are unable to penetrate far into the mucus, therefore tending to remain in or near the condensation layer, it is possible to estimate the concentration of particulate mass in comparison to the concentration of mucus solids. Concentrating the 100 μg

particulate mass in the 1 μm this condensation layer with 60 cm^2 of tracheal surface area gives a solids mass fraction of 0.6 — in comparison to the 0.05 solids mass fraction of hydrated mucus. Such particle coverage likely fouls the hydrogel, potentially retarding the movement of other, smaller particles, while decreasing overall water permeability of the mucus.

To characterize osmotic water flow in across airway mucus barriers, and begin to estimate impact of deposited airborne particle fouling, we modeled mucus as a porous medium with infinitely long cylindrical pores (i.e., the radius of the pores, *R,* much smaller than the length of the pores or the mucus thickness, *L* ~ 23 μm). Each pore is identical to the other, and of an approximate radius ~ 250 nm in hydrated and clean mucus, while with deposited particle clogging may be of a smaller effective radius. Steady-state diffusion of molecular and particulate osmolytes through the pores of the mucus gel leads to a steady mass flow of water (Q_m) in the opposite direction of the concentration gradient determined by (Anderson & Malone 1974)

$$Q_m = -\rho \sigma A P_m \Delta \Pi \tag{3}$$

where ρ is the mass density of water, and A the area of the upper airway compartment, with

$$\Delta \Pi = \sum_i n_i R_g T_k \Delta C_i + k T_k \Delta C_p \tag{4}$$

the osmotic pressure difference on either side of the membrane owing to concentration differences of osmolytically active molecules (*i*) in solution and Brownian particles (*p*). Here, R_g is the molar gas constant (8.315 J/K-mol), T_k the temperature (°K), k the Boltzmann constant (1.381 x 10^{-23} J/K) and, ΔC the concentration difference of all osmotic solutes across the mucus layer ($\Delta C = C_C — C_{PCL}$). The hydraulic membrane coefficient can be expressed as (Anderson & Malone 1974)

$$P_m = \epsilon \frac{R^2}{8\pi\mu L} \tag{5}$$

with ε the porosity of the membrane (0.95), μ = 0.01 g-s/cm, L = 23 μm. The particle/solute reflection coefficient through the porous hydrogel can be expressed per particle by

$$\sigma = \left(1 - \Phi^2\right)^2 \tag{6}$$

representing the degree to which solutes are prevented from entering the pores of the hydrogel, where

$$\Phi = 1 - \lambda \tag{7}$$

is the partition coefficient in the mucus membrane, and

$$\lambda = \frac{a}{R}$$

the ratio of osmolyte size to pore size. In the case where *a* is much smaller than *R*, as with salt ions or ultrafine particles, reflection can still occur owing to wall interactions with the solute. These can be characterized by an absorption potential *V* (Anderson & Malone 1974) and shown to have the following form in the case of $a << R$ (Anderson & Malone 1974)

$$\Phi \approx e^{-V} \tag{8}$$

Values of V far smaller than 1 reflect an essentially irreversible attraction between the solute and the mucus elements as ion association with charged mucin surfaces.

Relative Salt & Particle Osmotic Mucus Stress Contributions

To assess the relative importance of small particles and salt ions to the overall osmotic pressure force acting on mucus it is instructive to estimate the salt osmotic pressure from Eq (4) as $\Delta\Pi \sim$ 8.315 J/K-mol x 310 K x 0.3 osmoles/L or ~ 760,000 Pascals (Newton/m^2). Comparing this to the particle osmotic pressure acting in the very thin layer of water over mucus (the number of resident particles of 20 nm average diameter will be ~ 3 x 10^{12} or a condensation layer concentration of ~ 1/2 x $10^{21}/m^3$ assuming a 24 h clearance time) i.e., $\Delta\Pi \sim$ 1.381 x 10^{-23} J/K x 310 K x $10^{22}/m^3$ or ~ 20 Pascals (Newton/m^2) reveals salt ions to be the predominant contributor to osmotic stress on mucus in the airways. Both salt and particle contributions are overwhelmingly larger than predicted epithelial stress contributions (~ 1 Pa or less) of heat and momentum transfer owing to water evaporative and air flow stresses during normal tidal breathing (Wu et al 2015, 2018).

An estimate of the water permeability of mucus can therefore be determined from Eq (3) and using Eq (8) for the ion reflection coefficient with $V \sim (a/R)$ (using $a \sim 0.25$ nm and $R \sim 250$ nm gives $\sigma \sim 10^{-6}$) — note this value is approximately the same as the one deduced from Eq. (6) assuming merely steric hindrance as the two expressions become identical for very small λ —

$$P_m = 1.7 \times 10^{-4} \frac{m^2 s}{kg}$$

In conventional units we estimate the mucus water permeability to have a value of 2.4×10^{-2} m/s ($= \sigma P_m R_g T_k / v_w$ where $v_w = 18.14 \times 10^{-6}$ m^3/mol is the molar volume of water). Particle fouling of mucus (or any increase in solids content of mucus by particle fouling or dehydration) might render small particles a factor in the mucus permeability in two obvious ways. Deposited particles might reduce P_m by increasing steric hindrance. They might also be anticipated to increase the reflection coefficient. Recalling the 5 orders of magnitude difference in salt versus particle concentrations on the breathing of typical particle-laden air, the reflection coefficient would need to increase from 10^{-6} by one to three orders of magnitude — and the water permeability reduce by two to four orders of magnitude — for particles themselves to be direct and decisive contributors to the overall osmotic stress on the mucus, and in this case mucus is so impermeable to water that excessive dehydration of the upper airways is bound to occur.

Displacement of the Mucus Membrane

Displacement of mucus into the PCL by way of the osmotic stress accompanying water evaporation can be quantified by application of Newton's first law on the mucus mass M_m, yielding

$$d_{PCL} = u_m t + \frac{1}{2}\frac{\Delta\Pi}{M_m} A t^2 = l_{PCL}^0 - l_{PCL}$$

where $u_m = Q_m/\rho A$ is the velocity of the mucus hydrogel toward or away from the epithelium, t is time, and l_{PCL} is the thickness of the PCL. In steady-state conditions

$$d_{PCL} \approx u_m t \approx l_{PCL}^0 - l_{PCL} \qquad (9)$$

To further understand mucus displacement it is important to determine the degree to which water that evaporates from the upper airways is drawn from within the airway lining fluid by osmosis or is supplied by condensation on exhalation. Condensation rate in the upper airways on exhalation (Q_c) relates to evaporation rate on inhalation (Q_e) by the relative humidity of the exhaled air from beyond the carina (RH_{exh})

$$Q_c = \frac{RH_{exh}}{100} Q_e$$

The simplifying assumption that the relative humidity at the carina on inhalation RH_{inh} is maintained into the airways up to the airway generation where full saturation occurs RH_{exh} — can be stated as

$$\frac{RH_{exh}}{100} = \frac{1}{V_{exh}}[V_{sat}\frac{RH_{inh}}{100} + (V_{exh} - V_{sat})] \tag{10}$$

or

$$\frac{RH_{exh}}{100} = 1 - \chi \tag{11}$$

Here V_{sat} is the air volume from the carina to the airway generation where saturation occurs, and V_{exh} is the total volume of exhaled air — i.e., 1/2 liter in our example. The *airway condensation factor*

$$\chi = 0.66 + \frac{V_{sat}}{V_{exh}}(1 - \frac{RH_{inh}}{100}) \tag{12}$$

expresses the fractional degree to which evaporation from the upper airways is supplied by water drawn from the ALF versus water that has been condensed from lung air, the 0.66 factor reflecting that full consideration of heat and mass transfer (Haut et al 2021) indicates $\sim 33\%$ of water that is evaporated on inhalation is condensed on exhalation. Variations in χ from the average 33% replenishment of the condensation layer by exhaled air, i.e.

$$\xi = \frac{V_{sat}}{V_{exh}}(1 - \frac{RH_{inh}}{100})$$

are attributable to the degree to which exhaled air is less than fully saturated with water.

The displacement of the mucus membrane on inhalation by the amount d_{PCL} as indicated in Eq (9) is reversed on exhalation, so that the membrane vibrates with respect to the underlying layer of water. The mean displacement of the mucus membrane toward the epithelium many identical breath cycles is driven by the net time-averaged movement of water Q_m through the mucus following inhalation and exhalation, i.e., the difference between the rate of condensation and exhalation

$$\overline{Q_m} = \overline{Q_e} - \overline{Q_c} = \overline{Q_e}\chi$$

This relationship allows the time-averaged displacement of the mucus membrane to be estimated

$$\overline{l_{PCL}} = l^0_{PCL} - \overline{d}$$

$$\overline{l_{PCL}} = l^0_{PCL} - \frac{1}{2}\overline{Q_e}T\frac{\chi}{\rho A}$$

where total inhalation and exhalation times are identical, $T=T_{inh}=T_{exh}$. Mass conservation between the condensation layer, mucus layer, PCL and epithelium necessitates

$$\overline{Q_m} = \overline{Q_{epith}} = -\rho\sigma A\overline{P_m}\Delta\overline{\Pi_m} = -\rho\sigma A\overline{P_{epith}}\Delta\overline{\Pi_{epith}}$$

Here the over bar connotes time average and the (transcellular and paracellular) epithelial (epith) transport characteristics mirror those of the mucus itself. The osmotic pressure imbalances (ignoring particle effects) across the membrane and epithelium are related to the ion concentration differences produced by evaporation of water by

$$\Delta\overline{\Pi_m} = nR_gT_k(\overline{C_c} - \overline{C_m})$$

$$\Delta\overline{\Pi_{epith}} = nR_gT_k(\overline{C_{PCL}} - \overline{C_*})$$

where * denotes the surrounding tissues and cellular mass. We assume the hydration state of the tissues surrounding the airways to be relatively constant over the course of the breathing, such that the time averaged concentration of salt ions in the surrounding tissues is a constant C_*

$$\overline{C_*} = C_*$$

We also assume the mean time-steady state concentration of salt ions in the ALF is regulated by the epithelium to be constant, and that the mucus is undeformed (meaning a conservation of mass of osmotic solutes in the mucus), such that the total mass of salt ions is

$$\overline{M_{ALF}} = \overline{M_c} + \overline{M_{PCL}} + \overline{M_m} = (A\overline{l_c}\overline{C_c} + A\overline{l_{PCL}}\overline{C_{PCL}} + Al^0_m C_*) = (Al^0_c + Al^0_{PCL} + Al^0_m)C_*$$

and

$$\overline{C_m} = C^0_m = C_*$$

It follows that

$$\overline{C_c} = C_*\left(1 + \frac{\overline{Q}_e\chi}{\overline{PE_{epith}}} + \frac{\overline{Q}_e\chi}{\overline{PE_m}}\right) \tag{20}$$

$$\overline{C_{PCL}} = C_*\left(1 + \frac{\overline{Q}_e\chi}{\overline{PE_{epith}}}\right) \tag{21}$$

where

$$\overline{PE_m} = \rho\overline{\sigma_m}\overline{P_m}R_g T_k C_* \tag{22}$$

$$\overline{PE_{epith}} = \rho\overline{\sigma_{epith}}\overline{P_{epith}}R_g T_k C_* \tag{23}$$

Note that with the value determined above for $\sigma_m P_m \sim 10^{-10}$ m^2 s/kg (equal to a mucus water permeability in conventional units ~ 10^{-2} m/s) and with $\sigma_{epith} P_{epith} \sim 10^{-12}$ m^2 s/kg (given experimentally reported epithelial water permeabilities ~ 10^{-4} m/s) it follows that

$$\overline{PE_m} \approx 5 \times 10^2 mg/s \qquad \overline{PE_{epith}} \approx 5 \times 10^4 mg/s$$

From the above we have

$$\overline{l_c} = \frac{l_c^0 + l_{PCL}^0\left(1 - \frac{1}{2}\frac{\overline{Q_e}\chi T}{\rho A}\right)\left(1 + \overline{Q_e}\chi/\overline{PE}_{epith}\right)}{1 + \overline{Q_e}\chi/\overline{PE}_{epith} + \overline{Q_e}\chi/\overline{PE}_m} \quad (24)$$

In normal hydrated airways the membrane and epithelial permeation rates are relatively large, meaning they contribute in healthy circumstances only a minor amount to Eq. (24). Thus Eq. (24) reduces in normal circumstances to

$$\overline{l_c} = l_c^0 - \frac{1}{2}\frac{\overline{Q_e}\chi}{\rho A} \quad (25)$$

The Interrelated Thinning of the Condensation Layer and the PCL

The PCL thins in a very intuitive way. The contribution

$$\frac{1}{2}\overline{Q_e}T\frac{\chi}{\rho A} \quad (26)$$

in Eq. (25) represents the net water loss to the air from the condensation layer over a full cycle of breathing. The condensation layer meanwhile generally thickens in a parallel way to the PCL, while it being sensitive to the permeation of water through the mucus and epithelial membranes. Setting the left side of Eq (24) to zero leads to the definition of a critical epithelial permeability

$$\overline{PE_{epith}}^{CRIT} = \chi\overline{Q_e}\left(\frac{l_{PCL}^0 - \frac{1}{2}\frac{\overline{Q_e}\chi T}{\rho A}}{l_c^0 + \frac{1}{2}\frac{\overline{Q_e}\chi T}{\rho A}}\right) \quad (27)$$

characterizing a condition where the airway epithelium is unable to supply the water needed to hydrate inhaled air. The condensation layer in this case disappears. At values of epithelial permeability below the critical value (above) the condensation layer thickness becomes negative, and the water/air surface begins to recede into the mucus, with the mucus drying out. A similar

loss of condensation layer thickness can occur when the mucus permeability becomes vanishingly small, as on shrinkage of the mucus hydrogel (increase in solids content) during drying, acidification, and evolution of ionic composition. With low mucus permeability, the thickness of the condensation layer becomes vanishingly small, while it does not entirely disappear so long as epithelial permeation is sufficiently large.

ALF Dysfunction (Inflammatory Markers, CBF, EBP)

Quantitative estimates of airway dysfunction associated with small changes of mucus placement and ALF solute concentration relative to the fully hydrated PCL thickness and the isotonic salt ion concentration C_*, can be determined by perturbation analysis, i.e.,

$$\alpha \approx \alpha_0 + \delta\alpha_1 + \delta^2\alpha_2 + \delta^3\alpha_3 + \ldots \quad (28)$$

where α is a measure of dysfunction (as in the concentration of inflammatory marker in the ALF), α_0 is a hydrated equilibrium value, δ is a small dimensionless parameter reflecting the force of departure from the equilibrium state, and α_1, α_2, and α_3 are first, second and third order approximations of the variable α.

For dysfunction variables dependent on the compressive force or conformation change applied to cilia by the displacement of the mucus that occurs on dehydration, δ can be expressed as the steady-state displacement of the mucus layer relative to the thickness of fully hydrated PCL), leading to

$$\overline{CI} \approx CI_0[1 + \alpha_I \frac{\overline{Q_e} T \chi}{A}] \quad (29)$$

relative to a baseline value CI_0 and dimensional (cm^2/mg) inflammatory constant α_I. A similar expression follows for cilia beat frequency (CBF) relative to a hydrated state CBF_0 and dimensional (cm^2/mg) CBF constant α_{CBF}

$$\overline{CBF} \approx CBF_0[1 - \alpha_{CBF} \frac{\overline{Q_e} T \chi}{A}] \quad (30)$$

Loss of ALF volume, as occurs with inadequate permeation of mucus or epithelial layers, increases nonvolatile solute concentration in the ALF. The impact of ALF volume reduction on surfactants is to increase surfactant absorption onto the free ALF (condensation layer) surface. Breakup of the air-water interface can occur by the shear flow of air on inhalation or exhalation over the air-water surface, a phenomenon common in nature, as in the formation of sea spray at wind speeds exceeding approximately 2 m/s (Liu et al 2021). The instability of free liquid surfaces under the shear flow of air is well characterized in water (Newtonian) systems by the classical analysis of Rayleigh Taylor instability (Note 3, Main Article). Shear flow of air produces lateral stress on the air-water surface. Spatial variations of the air flow, as are inevitable in the natural flow of an air stream, produces spatial variations of the air-water shear stress, and this leads to wave formation, with compression of the surface happening in the troughs and valleys of the wave, and stretching occurring at the "saddle points" of the wave intermediate to the troughs and valleys. In the absence of surfactant, the natural surface tension of water (γ) provides a counteracting force on those regions of the surface that are stretched, pulling water out of the troughs and valleys, and thereby stabilizing the surface, resisting wave growth. Surfactant, which generally lowers surface tension, can both further stabilize and de-stabilize the surface against wave formation (and breakup) in the following way. In a system with a strong surfactant and slow absorption time scale relative to the stretching time scale of the surface, stretching of the surface reduces the surface tension in the compressed regions of the wave (troughs and valleys) while it increases the surface tension in the stretched regions of the wave; these phenomena stabilize the surface against wave formation and provide an underlying rationale for why surfactant systems tend to promote stable configurations (e.g., foams and emulsions) that are thermodynamically unstable. In the case where strong surfactant is present while with a very fast absorption time scale relative to the stretching time scale (as may occur when the bulk concentration of surfactant is significantly increased relative to a base state in which the surface is already saturated with surfactant), and where multiple surfactants are present with the most fast absorbing surfactants being the strongest, the stretching of the wave surface will lead to the more rapid absorption of the strongest surfactants, tending to diminish the surface tension precisely in those regions where stretching is occurring. This can lead to a gradient of tension favoring further growth of the crests and troughs of the wave, generating instability and breakup. This phenomenon is fundamental to strategies to "break" foam and emulsion systems by addition of a relatively strong surfactant.

The dependence of surface tension on surfactant concentration can be expressed in terms of a surface elasticity (Note 3, Main Article)

$$E_s = -\frac{\partial \gamma}{\partial C_s} \tag{31}$$

where C_s is the surface concentration of surfactant. High surface elasticity produces stable surfaces in the case of slow surfactant absorption relative to the time scale of wave stretching — and unstable surfaces in the case of fast absorption of mixed surfactant systems where the fastest absorbing surfactants are the strongest surfactants (Edwards et al 1991, Lucassen et al 1993). The ALF being a mixed surfactant system, with strong lung surfactants (e.g., DPPC) being produced predominantly in the alveolar region of the lungs, while also in the upper airways and undergoing transport up and down the airway tree on the exhalation and inhalation of air in the form of droplet nuclei and larger respiratory droplets. Increase in solute concentration within the ALF on the evaporation of water will increase surfactant concentration and favor absorption onto the shear-flow perturbed air-ALF surface of the strongest surfactants, such that instability and breakup of the surface of the ALF will grow with solute concentration.

Equation (31) can be used then to estimate surface breakup under the shear flow of air that occurs during inhalation. Assuming the number of droplets formed by the breakup of the condensation layer surface is proportional to the number of exhaled droplets in a clean-room environment (i.e., where all of the aerosol particles exhaled from the nose and mouth represent droplets formed within the airways), exhaled breath particles *EBP* on normal tidal breathing can be estimated to first-order approximation by a linear relation to condensation layer solute concentration relative to a hydrated state EBP_0, with α_{EBP} a dimensionless breakup constant

$$\overline{EBP} \approx EBP_0[1 + \alpha_{EBP}(\frac{\overline{Q_e}\chi}{PE_{epith}} + \frac{\overline{Q_e}\chi}{PE_m})] \quad (32)$$

On the Temporary Regulation of the Condensation Layer by Inhaled Salty Water

Depositing a volume of hypertonic saline droplets V_D on a region of the airways of total surface area *A* and with ALF water volume V_{ALF} alters the concentration of salt ions in the airways from C_* to C_+ by an amount

$$C_+ = \frac{C_* V_{ALF} + C_D V_D}{V_{ALF} + V_D} \quad (33)$$

Osmotic regulation of the ALF volume to equilibrate salt ion concentrations across the apical epithelial membrane results in a volume of water egress by osmosis V_{osm} of an amount

$$\frac{C_+}{C_*} = \frac{V_{ALF} + V_D + V_{osm}}{V_{ALF} + V_D} \tag{34}$$

Assuming the salt mass fraction in the deposited droplets and in the ALF is small compared to unity the water transport across the mucus post deposition and across the apical epithelium by osmosis largely remains above and beneath the mucus, such that the displacement of the mucus by an amount d_{osm} in steady state can be estimated by

$$\frac{C_+}{C_*} = \frac{V_{ALF} + V_D + V_{osm}}{V_{ALF} + V_D} \approx \frac{V_{PCL} + V_{osm}}{V_{PCL}} \approx \frac{\overline{l_{PCL}} + \overline{d}}{\overline{l_{PCL}}} \tag{35}$$

This gives

$$\overline{d_{osm}} \approx \overline{l_{PCL}}(\frac{C_+}{C_*} - 1) \tag{36}$$

From the above

$$\overline{d_{osm}} \approx \overline{l_{PCL}}[\frac{M_D(C_D - C_*)}{C_*(M_{ALF} + M_D)}] \tag{37}$$

where M_D and M_{ALF} are the masses of the deposited water and ALF respectively.

Finally, Table 1 (Main Article) provides estimates of the depth of air penetration and air volume (V_{sat}) needed to fully saturate inhaled air — using the Weibel airway geometry (Table S1) from the main bronchi to the lower airways. While the convective evaporation contribution to Eq (2) is negligible beyond the carina, significant evaporation can still occur in the central airways even in the ideal circumstances of equatorial (30^0C) air.

Generation	# Airways	Radius	Length	A	V_{sat}
1	2	0.61	4.8	37	11
2	4	0.41	1.9	9.6	15
3	8	0.28	0.8	11	17
4	16	0.30	1.3	39	23
5	32	0.18	1.1	40	26
6	64	0.14	0.9	51	33
7	128	0.12	0.8	77	38
8	256	0.09	0.6	87	42
9	512	0.08	0.5	129	47
10	1024	0.07	0.46	207	54
11	2048	0.06	0.39	301	63
12	4096	0.05	0.33	424	75
13	8192	0.04	0.27	556	85
14	16384	0.04	0.23	1152	108
15	32768	0.03	0.20	1235	123
16	65536	0.03	0.17	2099	155
17	131072	0.03	0.14	3457	207

Table S1. Weibel model human airway generation geometrical characteristics.

References

Anderson JL and Malone DM (1974) Mechanism of osmotic flow in porous membranes. *Biophysical Journal* 14(12), 957–982. https://doi.org/10.1016/S0006-3495(74)85962-X

Beverly S. Cohen , Robert G. Sussman & Morton Lippmann (1990) Ultrafine Particle Deposition in a Human Tracheobronchial Cast, Aerosol Science and Technology, 12:4, 1082-1091, DOI: 10.1080/02786829008959418

Darquenne, Chantal(2020). Deposition Mechanisms. Journal of Aerosol Medicine and Pulmonary Drug Delivery.181-185.http://doi.org/10.1089/jamp.2020.29029.cd

Edwards DA, Brenner H and Wasan DT (1991) Interfacial Transport Processes and Rheology. Boston, MA: Butterworth-Heinemann, 558 pp.

Ferron GA, Haider B and Kreyling WG (1985) A method for the approximation of the relative humidity in the upper human airways. *Bulletin of Mathematical Biology* 47, 565–589. https://doi.org/10.1016/S0092-8240(85)90022-9

Haut B, Nonclercq A, Buess A, Rabineau J, Rigaut C and Sobac B (2021) Comprehensive analysis of heat and water exchanges in the human lungs. *Frontiers in Physiology* 12, 649497. https://doi.org/10.3389/fphys.2021.649497

Hill DB, Button B, Rubinstein M, Boucher RC. Physiology and pathophysiology of human airway mucus. Physiol Rev. 2022 Oct 1;102(4):1757-1836. doi: 10.1152/physrev.00004.2021. Epub 2022 Jan 10. PMID: 35001665; PMCID: PMC9665957.

Liu S, Liu CC, Froyd KD, Schill GP, Murphy DM, Bui TP, Dean-Day JM, Weinzierl B, Dollner M, Diskin GS, Chen G and Gao RS (2021) Sea spray aerosol concentration modulated by sea surface temperature. *Proceedings of the National Academy of Sciences* 118(9), e2020583118. https://doi.org/10.1073/pnas.202058311

Lucassen-Reynders EH (1993) Interfacial viscoelasticity in emulsions and foams. *Food Structure* 12(1), 1.

McArthur AJ (1990) An accurate solution to the penman equation. *Agricultural and Forest Meteorology*, 51, 87–92.

Schuster BS, Suk JS, Woodworth GF, Hanes J. (2013) Nanoparticle diffusion in respiratory mucus from humans without lung disease. Biomaterials. Apr;34(13):3439-46. doi: 10.1016/j.biomaterials.2013.01.064. Epub 2013 Feb 4. PMID: 23384790; PMCID: PMC3590854.

Walji, Nimisha (2015) A Systematic Correlation of Nanoparticle Size with Diffusivity through Biological Fluids. PhD Thesis Imperial College (https://spiral.imperial.ac.uk/bitstream/10044/1/6080/1/Walji-N-2010-PhD-Thesis.pdf)

Wu D, Miyawaki S, Tawhai MH, Hoffman EA, Lin CL. A Numerical Study of Water Loss Rate Distributions in MDCT-Based Human Airway Models. Ann Biomed Eng. 2015 Nov;43(11):2708-21. doi: 10.1007/s10439-015-1318-3. Epub 2015 Apr 14. PMID: 25869455; PMCID: PMC4605848.

Wu D, Boucher RC, Button B, Elston T, Lin CL. (2018) An integrated mathematical epithelial cell model for airway surface liquid regulation by mechanical forces. J Theor Biol. 438:34-45. doi: 10.1016/j.jtbi.2017.11.010. Epub 2017 Nov 15. PMID: 29154907; PMCID: PMC5764545.
